# Medical Degrees and Diplomas

**Published:** 1898-09-03

**Authors:** 


ABERDEEN.

University of Aberdeen.

### MEDICAL DEGREES AND DIPLOMAS.

Medical degrees and diplomas are granted by the following bodies:—

In England.

The University of London (M.B., B.S., M.D., M.S.): Fee for the M.B., £17, including matriculation.

The University of Oxford (B.M. and B.Ch., which, however, are only conferred after taking the B.A., D.M., M.Ch.): Fee for B.M. and B.Ch., £14, in addition to fees for B.A.

The University of Cambridge (M.B., B.C., M.D., M.C.): The fees for the M.B. degree amount to about 12 guineas, in addition to those required for the Previous Examination.

The University of Durham (M.B., B.S., M.D., M.S.): Fees for M.B., £26.

The Victoria University (M.B., Ch.B., M.D., Ch.M.): Fees for M.B., £17.

The Conjoint Examining Board in England (M.R.C.S. and L.R.C.P.): Fee, 35 guineas.

The Royal College of Physicians (L.R.C.P., M.R.C.P., F.R.C.P.)

The Royal College of Surgeons (M.R.C.S., F.R.C.S.)

The Society of Apothecaries of London (L.S.A.): Fee, 15 guineas.

In Scotland.

University of Edinburgh, University of Glasgow, University of Aberdeen, University of St. Andrews. (The degrees granted by the Scottish Universities are M.B. and Ch.B., M.D., Ch.M., and the fees are practically the same, namely, about 22 guineas for the degrees M.B., Ch.B.)

The Royal College of Physicians of Edinburgh (L.R.C.P.Edin., M.R.C.P.Edin., F.R.C.P.Edin.)

The Royal College of Surgeons of Edinburgh (L.R.C.S.Edin., F.R.C.S.Edin.)

The Faculty of Physicians and Surgeons in Glasgow (L.F.P. and S. Glasgow).

(The latter three bodies co-operate in a conjoint examination, after passing which the student obtains the three diplomas, namely, L.R.C.P.Edin., L.R.C.S. Edin., and L.F.P.S. Glasgow. The fees for the triple diploma amount to £31.)

In Ireland.

University of Dublin (fee for the M.B., £17 5s.)

Royal University of Ireland (fee for the M.B., £17).

Royal College of Physicians of Ireland.

Royal College of Surgeons in Ireland.

(The Royal Colleges of Physicians and of Surgeons in Ireland have a conjoint examining board, which grants the diplomas L.R.C.P.I. and L.R.C.S.I. The fees amount to 42 guineas.)

Apothecaries' Hall of Ireland (fee for licence, 21 guineas).

